# A degenerate PCR-based strategy as a means of identifying homologues of aminoglycoside and β-lactam resistance genes in the gut microbiota

**DOI:** 10.1186/1471-2180-14-25

**Published:** 2014-02-05

**Authors:** Fiona Fouhy, R Paul Ross, Gerald F Fitzgerald, Catherine Stanton, Paul D Cotter

**Affiliations:** 1 Teagasc Food Research Centre, Moorepark, Fermoy, Cork, Ireland; 2 School of Microbiology, University College Cork, Cork, Ireland; 3 Alimentary Pharmabiotic Centre, Cork, Ireland

**Keywords:** Antibiotic resistance, Aminoglycosides, β-lactam, Gut microbiota, PCR

## Abstract

**Background:**

The potential for the human gut microbiota to serve as a reservoir for antibiotic resistance genes has been the subject of recent discussion. However, this has yet to be investigated using a rapid PCR-based approach. In light of this, here we aim to determine if degenerate PCR primers can detect aminoglycoside and β-lactam resistance genes in the gut microbiota of healthy adults, without the need for an initial culture-based screen for resistant isolates. In doing so, we would determine if the gut microbiota of healthy adults, lacking recent antibiotic exposure, is a reservoir for resistance genes.

**Results:**

The strategy employed resulted in the identification of numerous aminoglycoside (acetylation, adenylation and phosphorylation) and β-lactam (including *bla*_OXA_, *bla*_TEM_, *bla*_SHV_ and *bla*_CTX-M_) resistance gene homologues. On the basis of homology, it would appear that these genes originated from different bacterial taxa, with members of the *Enterobacteriaceae* being a particularly rich source. The results demonstrate that, even in the absence of recent antibiotic exposure, the human gut microbiota is a considerable reservoir for antibiotic resistance genes.

**Conclusions:**

This study has demonstrated that the gut can be a significant source of aminoglycoside and β-lactam resistance genes, even in the absence of recent antibiotic exposure. The results also demonstrate that PCR-based approaches can be successfully applied to detect antibiotic resistance genes in the human gut microbiota, without the need to isolate resistant strains. This approach could also be used to rapidly screen other complex environments for target genes.

## Background

Almost as soon as the widespread therapeutic use of antibiotics occurred, bacteria displaying diverse and complex mechanisms of resistance became problematic [1,2]. As the human gut is one of the most densely populated microbial environments, it has been postulated that it can act as a considerable reservoir for antibiotic resistance genes [3]. Thus, gut microbes may disseminate antibiotic resistance genes to other commensals or to bacteria transiently colonising the gut [4]. Given that antibiotics are known to exert significant and sustained negative effects on the gut microbiota [5,6], possessing resistance genes can provide a significant selective advantage to a subpopulation of microorganisms in individuals undergoing antibiotic treatment [7]. The aminoglycosides and β-lactams are two large families of antibiotics which are frequently employed in clinical settings. The aminoglycosides, which were first characterised in 1944, [8] function by binding to the 30S subunit of the prokaryotic ribosome resulting in disruption to protein synthesis. Resistance to aminoglycosides can be through reduced aminoglycoside uptake or enzymatic modification of the aminoglycoside through acetylation (AAC), adenylation (ANT) or phosphorylation (APH). β-lactam antibiotics include the penicillins and cephalosporins and inhibit bacteria through disruption of cell wall biosynthesis [9,10]. Resistance to β-lactams can be due to alterations to penicillin binding proteins or to the porins in the outer membrane (in Gram negative targets) or alternatively through the production of β-lactamases, which hydrolyse the eponymous β-lactam ring rendering the antibiotic inactive [11,12].

The question of the evolutionary origin of antibiotic resistance genes has been the subject of much attention [9,13,14]. For quite some time it was thought that resistance evolved following exposure of bacteria to new antibiotics [15]. However, it is now apparent that repositories of antibiotic resistance genes exist such that, following the development and application of new antibiotics, bacteria possessing or acquiring such genes will gain a selective advantage and thus resistance will increase over time [16,17]. Previous studies have employed PCR to detect resistance genes in specific pathogens [18,19], though studies employing PCR to detect resistance genes in complex microbial environments have been limited. In one instance, a PCR-based approach was used to investigate the prevalence of gentamycin resistance genes in resistant isolates from sewage, faeces (from cattle and chickens), municipal and hospital sewage water and coastal water [20]. The utilisation of a PCR approach in that instance resulted in the identification of diverse genes encoding gentamycin modifying enzymes from across a broad host range, thus demonstrating the suitability of a PCR-based approach to investigate resistance genes present in complex environments. However, the study did not investigate antibiotic resistance genes in human gut microbiota and, to our knowledge, to date no such PCR-based studies exist. Given these findings and other indications that there exist large natural pools of antibiotic resistance genes within complex microbial populations, it is likely that the human gut also contains many such genes. However, until now, PCR-based strategies to detect antibiotic resistance genes in the gut microbiota have involved an initial culture-based screen for resistant isolates, followed by subsequent PCR-based approaches to identify the associated resistance genes. This does not take into consideration the fact that the vast majority of gut microbes are not easily cultured [21], and thus antibiotic resistance genes from such microorganisms would typically be overlooked.

Here we utilise degenerate PCR primers to investigate the presence of β-lactam resistance genes and each of the three categories of aminoglycoside modifying enzymes within human metagenomic DNA and in doing so demonstrate that the human gut microbiota is a reservoir for antibiotic resistance genes. Additionally, we establish that a PCR-based approach allows the rapid detection of such genes in the complex gut microbiota environment, without the need for an initial isolation of strains.

## Methods

### Recruitment of volunteers

Forty adults were recruited and each provided written, informed consent for participation in this study. Approval for this trial was received from the Clinical Research Ethics Committee of the Cork Teaching Hospitals, Cork, Ireland. Volunteers were aged 28.8 ± 3.8 years, were free from gastrointestinal disorders and had not been treated with antibiotics in the 6 months prior to sample collection. Fresh faecal samples were collected and stored at −80°C until processed.

### DNA extraction

Stool samples were weighed, homogenised and due to the total volume provided by each individual, samples had to be pooled to achieve the required volume for our metagenomic DNA extraction protocol. To facilitate this, an equal volume (250 mg) from each individual was taken and pooled to form one sample, from which metagenomic DNA was extracted. The DNA extraction procedure used was optimised for total bacterial genomic DNA extraction from stool samples. The stool sample was homogenized in PBS and centrifuged at 1000 g × 5 mins and the supernatant was removed and retained. This was repeated 3 times. The supernatant then underwent Nycodenz (Axis Shield, UK) density gradient centrifugation separation, to separate out the bacterial cells from faecal matter. Following enzymatic lysis of bacterial cells using lysozyme and mutanolysin (Sigma Aldrich, Dublin, Ireland) protein precipitation using Proteinase K and ammonium acetate (Sigma Aldrich) was completed. Bacterial DNA was then precipitated and washed using standard chloroform and ethanol procedures. DNA was eluted in TE buffer.

### PCR-based detection of β-lactam resistance gene homologues

PCR-based detection of β-lactam resistance genes was completed using primers (MWG Eurofins, Germany) for the genes *bla*_TEM_[22,23], *bla*_SHV_[24] (both of which are classified as Bush group 2b β-lactamases), *bla*_CTX-M_[25] (an extended spectrum β-lactamase (ESBL) which confers resistance to cefotaxime), *bla*_OXA_[24] (ESBL, Bush group 2d ESBL) and *bla*_ROB_[23] (confers high level ampicillin resistance) (Table 1). PCRs were completed using bacterial metagenomic DNA and all PCRs were performed in triplicate. PCRs were completed on a G-storm PCR machine and for the primer sets *bla*_TEM_ primer set 1 (RH605/606), *bla*_TEM_ primer set 2 and *bla*_CTX-M_, PCRs were completed as previously outlined. For the primers *bla*_OXA_ and *bla*_ROB_ the PCR conditions were as follows: heated lid 110°C, 94°C × 5 mins followed by 30 cycles of 94°C × 30s, 64°C × 30s (*bla*_oxa_) or 62°C (*bla*_ROB_) and 72°C × 30s followed by 72°C × 10 mins and held at 4°C. For *bla*_SHV_ PCRs were performed as follows: heated lid 110°C, 94°C × 5 mins followed by 35 cycles of 94°C × 30s, 58°C × 30s and 72°C × 30s followed by a final extension step of 72°C × 10 mins and held at 4°C. All PCRs contained 25 μl Biomix Red (Bioline, UK), 1 μl forward primer (10pmol concentration), 1 μl reverse primer (10pmol concentration), metagenomic DNA (64 ng) and PCR grade water (Bioline, UK), to a final volume of 50 μl. Negative controls were completed for all primer sets. Gel electrophoresis was performed on all samples using 1.5% agarose gel in 1× TAE buffer.

**Table 1:** Primers used for the detection of β-lactamase and aminoglycoside resistant genes

**Location**	**Primer**	**Sequence 5′-3′**	**Amplicon Size (bp)**	**Annealing Temp°C**	**Source**
**β-lactamase genes**					
*Bla*_TEM_	RH605	TTTCGTGTCGCCCTTATTCC	692	60	Bailey *et al.* (2011) [22]
	RH606	CCGGCTCCAGATTTATCAGC			
	Bla_TEMF	TGGGTGCACGAGTGGGTTAC	526	57	Tenover *et al.* (1994) [23]
	Bla_TEMR	TTATCCGCCTCCATCCAGTC			
*Bla*_ROB_	Bla_ROBF	ATCAGCCACACAAGCCACCT	692	62	Tenover *et al.* (1994) [23]
	Bla_ROBR	GTTTGCGATTTGGTATGCGA			
*Bla*_SHV_	Bla_SHVF	CACTCAAGGATGTATTGTG	885	58	Briñas *et al.* (2002) [24]
	Bla_SHVR	TTAGCGTTGCCAGTGCTCG			
*Bla*_OXA_	Bla_OXAF	TTCAAGCCAAAGGCACGATAG	702	64	Briñas *et al.* (2002) [24]
	Bla_OXAR	TCCGAGTTGACTGCCGGGTTG			
*Bla*_CTX-M_	Bla_CTX-MF	CGTTGTAAAACGACGGCCAGTGAATGTGCAGYACCAGTAARGTKATGGC	600	55	Monstein *et al.* (2009) [25]
					
	Bla_CTX-MR	TGGGTRAARTARGTSACCAGAAYCAGCGG			
**AG resistant genes**					
*aac* (3)-I	Faac3-1	TTCATCGCGCTTGCTGCYTTYGA	239	58	Heuer *et al.* (2002) [20]
	Raac3-1	GCCACTGCGGGATCGTCRCCRTA			
*aac* (3)-II/VI	Faac3-2	GCGCACCCCGATGCMTCSATGG	189	58	
	Raac3-2	GGCAACGGCCTCGGCGTARTGSA			
	Facc3-6	GCCCATCCCGACGCATCSATGG			
	Raac3-6	CGCCACCGCTTCGGCATARTGSA			
*aac* (6′)-II/Ib	Faac6	CACAGTCGTACGTTGCKCTBGG	235	58	
	Raac6	CCTGCCTTCTCGTAGCAKCGDAT			
*ant* (2′)-I	Fant	TGGGCGATCGATGCACGGCTRG	428	58	
	Rant	AAAGCGGCACGCAAGACCTCMAC			
*aph* (2″)-I	Faphc	CCCAAGAGTCAACAAGGTGCAGA	527	55	
	Faphd	GGCAATGACTGTATTGCATATGA	572	55	
	Raph	GAATCTCCAAAATCRATWATKCC			
*aac* (6′)-*Ie*-*aph* (2″)-*Ia*	aac-aphF	GAGCAATAAGGGCATACCAAAAATC	505	47	*De Fatίma Silva Lopes et al.* (2003) [26]
	aac-aphR	CCGTGCATTTGTCTTAAAAAACTGG			
	aac6-aph2F	CCAAGAGCAATAAGGGCATACC	222	55	Schmitz *et al.* (1999) [27]
	aac6-aph2R	CACACTATCATAACCATCACCG			

*AG*: aminoglycoside. Type of gene i.e. beta-lactamase or AG given in bold.

### PCR-based detection of aminoglycoside resistance gene homologues

For the detection of aminoglycoside resistant genes, degenerate primer sets were used which had previously been designed and shown to amplify all known genes encoding gentamycin-modifying enzymes and similar, but as yet undiscovered, sequences [20]. PCRs were completed using primer sets (MWG Eurofins, Germany) for genes belonging to each group of aminoglycoside modifying enzymes namely, acetylation, adenylation and phosphorylation enzymes. DNA from positive controls (kindly gifted to us from the Smalla laboratory, JKI, Braunschweig) namely *Escherichia coli* S17-1 pAB2002 (*aac* (3)-Ia), *Pseudomonas aeruginosa* 88.341 F (*aac* (3)-Ib), *Enterobacter aerogenes* 17798 VDK (*aac* (3)-IIa), *E. coli* DH5α pSCH4203 (*aac* (3)-IIb), *E. coli* DH5α pSCH4101 (*aac* (3)-VIa), *P. aeruginosa* PST-1 (*aac* (3)-IIIa), *Acinetobacter baumannii* LBL.3 (*aac* (6′)-Ib), *P. aeruginosa* F-03 (*aac* (6′)-IIa), *E. coli* DH5α pSCH5102 (*aac* (6′)-IIb), *E. coli* CV600 pIE723 (*ant* (2″)-I), *E. coli* DH5α pAM6306 (*aph* (2″)-Ic) and *E. coli* NC95 (*aph* (2″)-Id) were used as positive controls for the PCR reactions. This ensured the specificity of the respective primer pairs. PCRs for the detection of acetylation genes *aac* (3)-I, *aac* (3)-II, *aac* (3)-III, *aac* (3)-VI and *aac* (6), adenylation genes *ant* (2″)-Ia and phosphorylation genes *aph* (2″)-Ic and *aph* (2″)-Id were completed as previously outlined [20] (Table 1). Additionally, PCRs using primers for the bifunctional gene *aac* (6″)-*Ie-aph* (2″) [26,27] (which encodes enzymes responsible for high level gentamycin resistance, as well as concomitant resistance to tobramycin and kanamycin) [27-31] were completed as follows: heated lid 110°C, 94°C × 5 mins followed by 30 cycles of 94°C × 30s, 47°C × 30s, 72°C × 30s, with a final extension step of 72°C × 10 mins and held at 4°C. All PCRs contained 25 μl Biomix Red (Bioline, UK), 1 μl forward primer (10pmol concentration), 1 μl reverse primer (10pmol concentration), metagenomic DNA (64 ng) and PCR grade water (Bioline, UK), to a final volume of 50 μl. Negative controls were run for all primer sets. All PCRs were performed in triplicate and analysed using gel electrophoresis, as described above.

### Cloning of PCR amplicons

Triplicate samples from successful PCR reactions were pooled and cleaned using AMPure magnetic bead-based PCR clean up kit (Beckman Coulter, UK). TOPO cloning reactions were performed on purified PCR products using the TOPO TA cloning kit (Invitrogen, Dublin, Ireland) to facilitate the sequencing of individual gene fragments. TOPO cloning reactions were then cloned into TOP10 *E. coli* (Invitrogen) as per the manufacturer’s instructions and plated onto LB (Difco) containing the appropriate antibiotic (either ampicillin 50 μg/ml or kanamycin 50 μg/ml; Sigma Aldrich, Dublin, Ireland) to select for the presence of the cloning vector. Transformants were selected from each TOPO cloning reaction and grown overnight in LB broth containing the suitable selective antibiotic (either ampicillin 50 μg/ml or kanamycin 50 μg/ml). Plasmids were extracted from overnight samples using QIAprep Spin Mini Prep kit (Qiagen, Sussex, UK) according to the manufacturer’s instructions and sent for Sanger sequencing (Source BioSciences, Dublin, Ireland).

### Bioinformatic analysis

Following Sanger sequencing, sequence reads were analysed using the NCBI protein database (BlastX; (http://blast.ncbi.nlm.nih.gov/)). In the event where multiple hits occurred, the BLAST hit which displayed greatest homology is reported.

## Results and discussion

### A PCR-based approach highlights the presence of β-lactamase gene homologues in the gut microbiota

The results of the β-lactamase-specific PCRs demonstrated the presence and diversity of class 2 β-lactamase genes in the gut microbiota of healthy adults (Table 2[32]). Of the β-lactam primers used, the primers designed to amplify *bla*_TEM_ genes yielded the greatest number of unique sequence hits (42% of selected TOPO sub-clones gave a unique hit). The majority of these genes exhibited a high percentage identity with genes from various members of the *Proteobacteria* including *E. coli*, *Klebsiella*, *Salmonella*, *Serratia, Vibrio parahaemolyticus* and *Escherichia vulneris*. The resistance of strains of *Salmonella* and *Serratia* to β-lactams via *bla*_TEM_ genes has been noted [33-35] and such strains have been associated with nosocomial infections [36]. In contrast, there have been relatively few studies of *bla*_TEM_ genes in *Vibrio parahaemolyticus* and *Escherichia vulneris*[37,38]. The identification of genes homologous to those from *Enterobacteriaceae* is not surprising given the prevalence of resistance genes among members of this family [12]. It was notable that the *bla*_TEM_ primers also amplified genes that resembled *bla*_TEM_ genes from some more unusual sources, including two genes from uncultured bacteria and from a Sar 86 cluster (a divergent lineage of γ-*Proteobacteria*) bacteria. This approach can thus provide an insight into possible novel/unusual sources of resistance genes, including those that culture-based approaches would fail to detect. Such results also highlight that had initial screening for resistant isolates been completed prior to PCR amplification of the resistance genes, such unusual sources of resistance genes may have been overlooked. Additionally, genes encoding ESBLs, including *bla*_TEM-116_, *bla*_TEM-195_ and *bla*_TEM-96_ amongst others, were also identified, with their closest homologues being members of the *Proteobacteria* (Table 2).

**Table 2:** Homologues of β-lactamase genes detected in the human gut microbiota via PCR techniques

**Accession #**	**Gene description**	**Closest homologue**	**E value**	**% identity**
***Bla***_**TEM**_				
ADE18890.1	β-lactamase TEM-1	*S. enterica* subsp. *enterica*	5e^-154^	99
AAS46844.1	β-lactamase TEM-1	*S. marcescens*	2e^-156^	100
AEN02824.1	β-lactamase TEM-1	*K. pneumoniae*	3e^-111^	99
AEN02817.1	β-lactamase TEM-1	*K. pneumoniae*	1e^-113^	99
ACV88636.1	β-lactamase TEM-1	*E. coli*	2e^-151^	99
AEL87577.1	ES β-lactamase TEM-116	*Vibrio parahaemolyticus*	5e^-154^	99
AEQ55231.1	β-lactamase TEM-1	*E. coli*	1e^-35^	45
ABQ14376.1	β-lactamase	*Uncultured soil bacterium*	6e^-05^	83
ADN79104.1	β-lactamase TEM	*Escherichia vulneris*	1e^-15^	86
WP_010157942.1	β-lactamase TEM	*Sar 86 cluster bacterium*	9e^-122^	83
ACI29961.1	β-lactamase TEM-1	*E. coli*	2e^-153^	99
AEQ39590.1	β-lactamase TEM-195	*E. coli*	5e^-93^	96
AAM22276.1	β-lactamase TEM-96	*E. coli*	7e^-139^	94
WP_019405145.1	β-lactamase TEM	*K. pneumoniae*	4e^-155^	99
AEW28787.1	β-lactamase TEM-1	*Uncultured bacterium*	1e^-133^	100
ABY81267.1	β-lactamase	*E. coli*	4e^-156^	100
AAF74292.1	ES β-lactamase	*E. coli*	5e^-155^	99
AFU53026.1	KPC-2 β lactamase	*S. marcescens*	2e^-112^	98
ADE18896.1	β-lactamase TEM-1	*Salmonella enterica*	2e^-113^	99
AEN02826.1	β-lactamase TEM-1	*K. pneumoniae*	4e^-113^	99
***Bla***_**ROB**_				
YP_252228.1	Hypothetical protein SH0313	*S. haemolyticus*	2e^-33^	44
***Bla***_**SHV**_				
WP_009348253.1	Hypothetical protein HMPREF 9332	*Alloprevotella rava*	3e^-07^	56
WP_017896153.1	β-lactamase	*K. pneumoniae* subsp. *pneumoniae*	0.0	99
WP_008157744.1	Hypothetical protein HMPREF 1077	*Parabacteroides johnsonii*	1.5	29
CAJ47138.2	β-lactamase	*K. pneumoniae*	0.0	99
ADU15837.1	BlaSHV132	*K. pneumoniae*	0.0	99
AEK80394.1	β-lactamase SHV140	*K. pneumoniae*	0.0	99
ABS72351.1	β-lactamase SHV103	*K. pneumoniae*	0.0	99
AAP03063.1	β-lactamase SHV48	*K. pneumoniae*	0.0	99
AEG79634.1	ES β-lactamase SHV120	*E. coli*		99
***Bla***_**CTX-M**_				
ABG46354.1	ES β-lactamase	*E. coli*	3e^-139^	99
AEZ49563.1	β-lactamase CTX-M-1	*E. coli*	2e^-138^	99
AEZ49551.1	β-lactamase CTX-M-1	*K. pneumoniae*	1e^-139^	100
ABG46356.1	ES β-lactamase	*K. pneumoniae*	9e^-139^	97
ABW06480.1	ES β lactamase CTX-M-15	*K. pneumoniae*	6e^-51^	94
AAB22638.1	β-lactamase penicillin hydrolase	*E. coli*	9e^-140^	100
BAD16611.1	β-lactamase CTX-M-36	*E. coli*	8e^-139^	99
YP_003717483.1	β-lactamase	*E. coli*	2e^-139^	100
ABN09669.1	β-lactamase CTX-M-61	*S. enterica*	2e^-138^	100

*ESBL*: extended spectrum β-lactamase. Gene names are in bold.

Using the *bla*_SHV_ primers, multiple genes sharing homology with genes from members of the *Enterobacteriaceae*, and *Klebsiella* and *E. coli* in particular were detected. In addition, amplicons with low percentage identity to genes from *Alloprevotella rava* and *Parabacteroides johnsonii*, respectively, were also identified. This is again consistent with existing research which states that *Enterobacteriaceae* are the primary source of *bla*_SHV_ genes [39-43]. Furthermore, the amplicons sequenced resembled various different types of ESBL-encoding SHV genes, including *bla*_SHV-132_, *bla*_SHV-140_ and *bla*_SHV-48_, thus again highlighting the genuine degeneracy of the primers used.

Additional PCRs were completed to identify other ESBLs, specifically CTX-M- and OXA-type β-lactamases (Table 2). A number of different CTX-M β-lactamases were detected, including CTX-M-1, CTX-M-15 and CTX-M-36. The fact that many of the β-lactamase genes detected using our approach share homology with resistance genes found in members of the phylum *Proteobacteria* is not surprising as, despite being typically less common than the *Bacteroidetes* or *Firmicutes* in the gut microbiota of healthy adults [21], members of this genus have been identified as sources of antibiotic resistance genes and have been frequently associated with nosocomial infections and outbreaks [36,39,44,45]. In the 1990s, TEM- and SHV-type ESBLs were the β-lactamases most frequently observed among *Enterobacteriaceae*[18]. However, more recently, CTX-M-type ESBLs have spread rapidly and are now the most prevalent ESBL in *Enterobacteriaceae* in several parts of the world [46]. In a recent report on antibiotic resistance threats in the USA, the Centre for Disease Control stated that ESBL-producing *Enterobacteriaceae* were a serious public health threat [47]. The report estimates that 26,000 infections and 1,700 deaths that occur each year in the United States are attributable to ESBLs and that upwards of 140,000 health-care related *Enterobacteriaceae* infections occur annually. Therefore the detection of homologues of ESBL-encoding genes in the gut microbiota of healthy individuals is significant and provides evidence of the ubiquitous nature of these resistance genes, even in the absence of recent antibiotic exposure. With respect to the CTX-M-type ESBLs, it is particularly notable that homologues of the *bla*_CTX-M-15_ gene were detected, as these have received significant attention due to their recent rapid spread and their association with multi-drug resistant *E. coli* responsible for outbreaks of antibiotic resistant infections [48,49]. In such cases, these genes have been found on multi-drug resistance-encoding regions of plasmids, thus facilitating the rapid transfer of these genes. The presence of such genes within the gut microbiota raises concerns that horizontal gene transfer may occur between commensals or to bacteria passing through the gut. If the resistance genes detected in our study are, or were to become, mobile, it would enable the gut to act not only as a source of resistance genes, but also as a site of resistance gene transfer. Although outside the scope of this study, studies investigating whether these genes are located on or near mobile genetic elements would be pertinent to ascertain the risk of the gut acting as a site for horizontal gene transfer.

When the *bla*_ROB_ primer set was employed to detect the presence of homologues of these ampicillin resistance-encoding genes, all amplicons sequenced were identical and shared 44% identity to *Staphylococcus haemolyticus bla*_ROB_ gene. Finally, this study did not detect *bla*_OXA_ gene homologues in our metagenomic sample. These findings are unexpected and may have occurred as a result of the particular affinity of the primer sets used.

### A PCR-based approach highlights the presence of aminoglycoside resistance encoding gene homologues in the gut microbiota

Degenerate primers were selected that amplify genes encoding aminoglycoside modifying enzymes from each of the enzyme modification groups, namely acetylation, adenylation and phosphorylation [32]. When primers were applied to detect acetylation-associated genes, it was established that the primers designed to target *aac* (3)-I, *aac* (3)-II, and *aac* (3)-III homologues did not generate amplicons. In each of these PCR reactions the positive controls successfully amplified, thus we are satisfied that the lack of amplification products for our metagenomic sample is a true result. However, a number of distinct *aac* (6) and *aac* (3)-VI homologues were detected and were found to resemble genes from a variety of genera, including *Acinetobacter*, *Pseudomonas* and *Enterobacter* (Table 3). The presence of aminoglycoside acetylation genes within these genera has been noted previously [50-53]. The detection of resistance genes resembling those seen in *A. baumannii* is a concern, as many strains of this species have been shown to exhibit multi-drug resistance [54,55]. In addition, homologues of genes from *Collinsella* and *Salmonella* were also detected. Primers designed to amplify bifunctional *aac* (6′)-*Ie*-*aph* (2′) genes were also employed. Our investigations revealed the presence of homologues of such genes, resembling those from *S. aureus*, *E. faecium* and *S. epidermidis*, all of which are known sources of these genes [27,56,57].

**Table 3:** Homologues of aminoglycoside resistance genes detected in the human gut microbiota via PCR techniques

**Accession #**	**Gene description**	**Closest homologue**	**E value**	**% identity**
***aac *** **(6)**				
AAA25680.1	AG 6′-N-acetyltransferase	*Pseudomonas fluorescens*	4 e^-48^	98
WP_006234103.1	Hypothetical protein Colaer00186	*Collinsella aerofaciens*	0.0	95
AAS45464.1	6′-N-acetyltransferase	*A. baumannii*	3e^-33^	75
***aac *** **(6′)-*Ie-aph *** **(2″)**				
WP_002304968.1	Phosphotransferase	*E. faecium*	9e^-108^	100
WP_001028140.1	Acetyltransferase GNAT	*S. aureus*	1e^-107^	99
WP_001028143.1	Acetyltransferase GNAT	*S. aureus*	1e^-107^	99
WP_010729367.1	Bifunctional AAC/APH partial sequence	*E. faecium*	5e-^106^	99
AAX82584.1	Bifunctional AG modifying enzyme	*Enterococcus faecalis*	2e^-112^	100
WP_002417297.1	6′ AG acetyltransferase	*E. faecalis*	3e^-111^	97
AFR11868.1	Bifunctional AG 6′-N acetytransferase/2′-AG phosphotransferases	*S. epidermidis*	1e^-43^	99
AFM29914.1	Gentamycin resistance protein	*Enterococcus* sp.	7e^-45^	97
***aph *** **(2″) Id**				
3SG8_A	Chain A crystal structure AG 2′ phosphotransferases	*E. casseliflavus*	1e^-110^	98
3N4T_A	Aph2″ chain a	*E. casseliflavus*	2e^-110^	99
AAT77696.1	AG modifying enzyme	*E. faecium*	1e^-68^	94
***Aph *** **(2″)-Ic**				
3TDVA	AG phosphotransferase	*Enterococcus gallinarum*	2e^-83^	97
***ant *** **(2″) Ia**				
YP_005176240.1	AG 2′–O-adenyltransferase	*Pasturella mutocida*	2e^-97^	100
WP_000314377.1	2′ AG nucleotidlytransferase	*A. baumannii*	3e^-94^	99
WP_000946493.1	2′ AG	*A. baumannii*	1e^-94^	99
ACJ47203.1	AG adenyltransferase	*E. coli*	6e^-94^	99
ACA48663.14	AG adenyltransferase	*Morganella morganii*	2e^-96^	99
***aac*** **(3)-VI**				
AAA16194.1	aac 3–6	*Enterobacter cloacae*	2e^-05^	77
WP_001642188.1	AG acetyltransferase	*S. enterica* subsp *enterica*	2e^-20^	98

*AG*: aminoglycoside. Gene names are in bold.

Homologues of aminoglycoside phosphorylation-encoding genes were also detected using a PCR-based approach, with both *aph* (2″)-Ic and *aph* (2″)-Id like genes being detected. These genes shared homology with genes from *Enterococcus* species, including *E. faecium* and *E. casseliflavus.* Aminoglycoside resistant *E. faecium* have received significant attention due to their role in nosocomial infections [58,59]. Notably, the role of mobile genetic elements in the maintenance and dissemination of multi-drug resistance in *Enterococcus faecalis* and *E. faecium* has previously been highlighted [30,60,61]. While it is not certain that the genes identified in this study are also associated with mobile elements, the possibility that resistance genes could be transferred to commensals is a concern. Homologues of aminoglycoside adenylation genes, *ant* (2″)-Ia, were also successfully detected. These resembled genes from *Pasteurella*, *Acinetobacter* and *E. coli* (Table 3), and the findings are thus consistent with previous research showing that these genes are most frequently detected in Gram negative bacteria [62]. Overall, the results demonstrate that the gut microbiota is a source of diverse aminoglycoside and β-lactam resistance genes, despite having had no recent antibiotic exposure. If these genes are expressed there is the potential that if antibiotic exposure occurred, bacteria containing these resistance genes would become the dominant component of the gut microbiota, as has been shown in previous studies [5,63].

## Conclusions

This study has highlighted the merits of applying a PCR-based approach to detect antibiotic resistance genes within the human gut microbiome. The results clearly demonstrate that the human gut microbiota is a considerable reservoir for resistance genes. Further studies are required to determine the exact sources of these genes and to determine if they have the potential to become mobile. Additionally, we have highlighted the successful application of a PCR-based screen of a complex environment without prior isolation of resistant isolates. The possibility exists to couple this approach with lower throughput next generation sequencing strategies, such as that provided by the Ion PGM 314 chip, in instances where great diversity is likely. Our approach could also be used in conjunction with functional screening of metagenomic libraries to enable the detection of genes present in a complex environment at a low threshold and that may have avoided capture in the metagenomic library, as shown in a recent study [64]. Such a PCR-based approach is not being proposed as a substitute for ultra-deep high-throughput shotgun sequencing of metagenomic DNA, rather it is a lower cost, more targeted, alternative which facilitates the detection and *in silico* analysis of specific gene sets of interest. Finally, while this study demonstrates that the gut microbiota is a source of diverse resistance genes, further studies are required to investigate the exact sources of these genes, their expression and whether they have the potential to become mobile. As the scientific community continues to gain knowledge with respect to the genetic mechanisms involved in providing resistance to various antibiotics, the design of additional sets of degenerate primers will be possible and will provide further opportunities for the use of PCR to rapidly and efficiently detect antibiotic resistance genes in complex microbial environments, including the human gut microbiota.

### Availability of supporting data

The data sets supporting results of this article are available in the LabArchives repository, [http://dx.doi.org/10.6070/H42V2D1V].

## Abbreviations

AAC: Acetylation enzymes; APH: Phosphorylation enzymes; ANT: Adenylation enzymes; ESBL: Extended spectrum β-lactamase; AG: Aminoglycoside.

## Competing interests

The authors declare that they have no competing interests.

## Authors’ contributions

FF conceived the study, was involved in the study design, performed the laboratory experiments and analysis and wrote the manuscript. RPR was involved in the study design and the drafting of the manuscript. GFF was involved in drafting of the manuscript. CS was involved in the study design and drafting of the manuscript. PDC conceived the study, was involved in the study design, interpretation of the data and drafting of the manuscript. All authors read and approved the final manuscript.

## References

[B1] Davies J Davies D Origins and evolution of antibiotic resistance Microbiol Mol Biol Rev 2010 74 417 433 10.1128/MMBR.00016-10 20805405 PMC2937522

[B2] Abraham E Chain E An enzyme from bacteria able to destroy penicillin Nature 1940 146 837 837 3055168

[B3] Salyers AA Gupta A Wang Y Human intestinal bacteria as reservoirs for antibiotic resistance genes Trends Microbiol 2004 12 412 416 10.1016/j.tim.2004.07.004 15337162

[B4] Broaders E Gahan CG Marchesi JR Mobile genetic elements of the human gastrointestinal tract: potential for spread of antibiotic resistance genes Gut microbes 2013 4 271 280 10.4161/gmic.24627 23651955 PMC3744512

[B5] Dethlefsen L Huse S Sogin ML Relman DA The pervasive effects of an antibiotic on the human gut microbiota, as revealed by deep 16S rRNA sequencing PLoS Biol 2008 6 e280 210.137/journal.pbio.0060280 10.1371/journal.pbio.0060280 19018661 PMC2586385

[B6] Cotter P Stanton C Ross R Hill C The impact of antibiotics on the gut microbiota as revealed by high throughput DNA sequencing Discov Med 2012 13 193 199 22463795

[B7] Sommer MOA Dantas G Church GM Functional characterization of the antibiotic resistance reservoir in the human microflora Sci 2009 325 1128 1131 10.1126/science.1176950 PMC4720503 19713526

[B8] Mingeot-Leclercq MP Glupczynski Y Tulkens PM Aminoglycosides: activity and resistance Antimicrob Agents Chemother 1999 43 727 737 10103173 10.1128/aac.43.4.727 PMC89199

[B9] Page MGP Beta-Lactam Antibiotics Antibiot Discov Dev 2012 1 79 117

[B10] Tipper DJ Strominger JL Mechanism of action of penicillins: a proposal based on their structural similarity to acyl-D-alanyl-D-alanine Proc Natl Acad Sci U S A 1965 54 1133 1141 10.1073/pnas.54.4.1133 5219821 PMC219812

[B11] Bush K Characterization of beta-lactamases Antimicrob Agents Chemother 1989 33 259 263 10.1128/AAC.33.3.259 2658779 PMC171476

[B12] Bush K Alarming beta-lactamase-mediated resistance in multidrug-resistant Enterobacteriaceae Curr Opin Microbiol 2010 13 558 564 10.1016/j.mib.2010.09.006 20920882

[B13] Kotra LP Mobashery S β-Lactam antibiotics, β-lactamases and bacterial resistance Bull Inst Pasteur 1998 96 139 150 10.1016/S0020-2452(98)80009-2

[B14] Tipper D Mode of action of β-lactam antibiotics Rev Infect Dis 1979 1 39 53 10.1093/clinids/1.1.39 400939

[B15] Hughes VM Datta N Conjugative plasmids in bacteria of the ‘pre-antibiotic’ era Nature 1983 302 725 726 10.1038/302725a0 6835408

[B16] Bhullar K Waglechner N Pawlowski A Koteva K Banks ED Johnston MD Barton HA Wright GD Antibiotic resistance is prevalent in an isolated cave microbiome PLoS ONE 2012 7 e34953 10.1371/journal.pone.0034953 22509370 PMC3324550

[B17] D’Costa VM King CE Kalan L Morar M Sung WWL Schwarz C Froese D Zazula G Calmels F Debruyne R Antibiotic resistance is ancient Nature 2011 477 457 461 10.1038/nature10388 21881561

[B18] Dallenne C Da Costa A Decré D Favier C Arlet G Development of a set of multiplex PCR assays for the detection of genes encoding important β-lactamases in Enterobacteriaceae J Antimicrob Chemother 2010 65 490 495 10.1093/jac/dkp498 20071363

[B19] Vannuffel P Gigi J Ezzedine H Vandercam B Delmee M Wauters G Gala J-L Specific detection of methicillin-resistant Staphylococcus species by multiplex PCR J Clin Microbiol 1995 33 2864 2867 8576335 10.1128/jcm.33.11.2864-2867.1995 PMC228596

[B20] Heuer H Krögerrecklenfort E Wellington E Egan S Elsas J Overbeek L Collard JM Guillaume G Karagouni A Nikolakopoulou T Gentamicin resistance genes in environmental bacteria: prevalence and transfer FEMS Immunol Med Microbiol 2002 42 289 302 10.1111/j.1574-6941.2002.tb01019.x 19709289

[B21] Eckburg PB Bik EM Bernstein CN Purdom E Dethlefsen L Sargent M Gill SR Nelson KE Relman DA Diversity of the human intestinal microbial flora Science 2005 308 1635 1638 10.1126/science.1110591 15831718 PMC1395357

[B22] Bailey JK Pinyon JL Anantham S Hall RM Distribution of the blaTEM gene and blaTEM-containing transposons in commensal Escherichia coli J Antimicrob Chemother 2011 66 745 751 10.1093/jac/dkq529 21393132

[B23] Tenover FC Huang MB Rasheed JK Persing DH Development of PCR assays to detect ampicillin resistance genes in cerebrospinal fluid samples containing Haemophilus influenzae Eur J Clin Microbiol 1994 32 2729 2737 10.1128/jcm.32.11.2729-2737.1994 PMC264151 7852564

[B24] Briñas L Zarazaga M Sáenz Y Ruiz-Larrea F Torres C β-Lactamases in ampicillin-resistant Escherichia coli isolates from foods, humans, and healthy animals Antimicrob Agents Chemother 2002 46 3156 3163 10.1128/AAC.46.10.3156-3163.2002 12234838 PMC128764

[B25] Monstein H-J Tärnberg M Nilsson LE Molecular identification of CTX-M and blaOXY/K1 β-lactamase genes in Enterobacteriaceae by sequencing of universal M13-sequence tagged PCR-amplicons BMC Infect Dis 2009 9 7 15 10.1186/1471-2334-9-7 19161622 PMC2651175

[B26] De Fátima Silva Lopes M Ribeiro T Abrantes M Figueiredo Marques JJ Tenreiro R Crespo MTB Antimicrobial resistance profiles of dairy and clinical isolates and type strains of enterococci Int J Food Microbiol 2005 103 191 198 10.1016/j.ijfoodmicro.2004.12.025 16083821

[B27] Schmitz F-J Fluit AC Gondolf M Beyrau R Lindenlauf E Verhoef J Heinz H-P Jones ME The prevalence of aminoglycoside resistance and corresponding resistance genes in clinical isolates of staphylococci from 19 European hospitals J Antimicrob Chemother 1999 43 253 259 10.1093/jac/43.2.253 11252331

[B28] Matsumura M Katakura Y Imanaka T Aiba S Enzymatic and nucleotide sequence studies of a kanamycin-inactivating enzyme encoded by a plasmid from thermophilic bacilli in comparison with that encoded by plasmid pUB110 J bacteriol 1984 160 413 420 6090428 10.1128/jb.160.1.413-420.1984 PMC214734

[B29] Ubukata K Yamashita N Gotoh A Konno M Purification and characterization of aminoglycoside-modifying enzymes from Staphylococcus aureus and Staphylococcus epidermidis Antimicrob Agents Chemother 1984 25 754 759 10.1128/AAC.25.6.754 6331299 PMC185635

[B30] Hegstad K Mikalsen T Coque T Werner G Sundsfjord A Mobile genetic elements and their contribution to the emergence of antimicrobial resistant Enterococcus faecalis and Enterococcus faecium Clin Microbiol Infect 2010 16 541 554 10.1111/j.1469-0691.2010.03226.x 20569265

[B31] Ferretti JJ Gilmore K Courvalin P Nucleotide sequence analysis of the gene specifying the bifunctional 6′-aminoglycoside acetyltransferase 2″-aminoglycoside phosphotransferase enzyme in Streptococcus faecalis and identification and cloning of gene regions specifying the two activities J bacteriol 1986 167 631 638 3015884 10.1128/jb.167.2.631-638.1986 PMC212936

[B32] Fouhy F Ross RP Fitzgerald GF Stanton C Cotter PD PCR sequencing data of aminoglycoside and beta-lactam resistance genes BMC microbiology 2013 http://dx.doi.org/10.6070/H42V2D1V; 2013 10.1186/1471-2180-14-25 PMC3917905 24499167

[B33] Morris D Whelan M Corbett-Feeney G Cormican M Hawkey P Li X Doran G First Report of Extended-Spectrum-β-Lactamase-Producing Salmonella enterica Isolates in Ireland Antimicrob Agents Chemother 2006 50 1608 1609 10.1128/AAC.50.4.1608-1609.2006 16569897 PMC1426976

[B34] Perilli M Felici A Franceschini N De Santis A Pagani L Luzzaro F Oratore A Rossolini GM Knox JR Amicosante G Characterization of a new TEM-derived beta-lactamase produced in a Serratia marcescens strain Antimicrob Agents Chemother 1997 41 2374 2382 9371336 10.1128/aac.41.11.2374 PMC164131

[B35] Zhao W-H Hu Z-Q Chen G Matsushita K Fukuchi K Shimamura T Characterization of imipenem-resistant Serratia marcescens producing IMP-type and TEM-type beta-lactamases encoded on a single plasmid Microbiol Res 2007 162 46 52 10.1016/j.micres.2006.06.005 16875810

[B36] Morosini MI Canton R Martinez-Beltran J Negri MC Perez-Diaz JC Baquero F Blazquez J New extended-spectrum TEM-type beta-lactamase from Salmonella enterica subsp. enterica isolated in a nosocomial outbreak Antimicrob Agents Chemother 1995 39 458 461 10.1128/AAC.39.2.458 7726515 PMC162560

[B37] Wong MHY Liu M Wan HY Chen S Characterization of Extended-Spectrum-β-Lactamase-Producing Vibrio parahaemolyticus Antimicrob Agents Chemother 2012 56 4026 4028 10.1128/AAC.00385-12 22508301 PMC3393472

[B38] Mokracka J Koczura R Kaznowski A Multiresistant Enterobacteriaceae with class 1 and class 2 integrons in a municipal wastewater treatment plant Water Res 2012 46 3353 3363 10.1016/j.watres.2012.03.037 22507248

[B39] Coque TM Oliver A Pérez-Díaz JC Baquero F Cantón R Genes Encoding TEM-4, SHV-2, and CTX-M-10 Extended-Spectrum β-Lactamases Are Carried by Multiple Klebsiella pneumoniae Clones in a Single Hospital (Madrid, 1989 to 2000) Antimicrob Agents Chemother 2002 46 500 510 10.1128/AAC.46.2.500-510.2002 11796363 PMC127031

[B40] Paterson DL Hujer KM Hujer AM Yeiser B Bonomo MD Rice LB Bonomo RA Extended-spectrum β-lactamases in Klebsiella pneumoniae bloodstream isolates from seven countries: dominance and widespread prevalence of SHV-and CTX-M-type β-lactamases Antimicrob Agents Chemother 2003 47 3554 3560 10.1128/AAC.47.11.3554-3560.2003 14576117 PMC253771

[B41] Heritage J M’Zali FH Gascoyne-Binzi D Hawkey PM Evolution and spread of SHV extended-spectrum β-lactamases in Gram-negative bacteria Journal of antimicrobial chemotherapy 1999 44 309 318 10.1093/jac/44.3.309 10511397

[B42] Babini GS Livermore DM Antimicrobial resistance amongst Klebsiella spp. collected from intensive care units in Southern and Western Europe in 1997–1998 J Antimicrob Chemother 2000 45 183 189 10.1093/jac/45.2.183 10660500

[B43] Pitout J Sanders C Sanders W Jr Antimicrobial resistance with focus on beta-lactam resistance in gram-negative bacilli Am J Med 1997 103 51 59 10.1016/S0002-9343(97)00044-2 9236486

[B44] Bonnet R Growing group of extended-spectrum β-lactamases: the CTX-M enzymes Antimicrob Agents Chemother 2004 48 1 14 10.1128/AAC.48.1.1-14.2004 14693512 PMC310187

[B45] Pitout JDD Laupland KB Extended-spectrum [beta]-lactamase-producing Enterobacteriaceae: an emerging public-health concern Lancet Infect Dis 2008 8 159 166 10.1016/S1473-3099(08)70041-0 18291338

[B46] Coque T Baquero F Canton R Increasing prevalence of ESBL-producing Enterobacteriaceae in Europe Euro Surveillance 2008 13 19 29 19021958

[B47] CDC Antibiotic resistance threats in the United States 2013 Retrieved from http://www.cdc.gov/drugresistance/threat-report-2013/

[B48] Boyd DA Tyler S Christianson S McGeer A Muller MP Willey BM Bryce E Gardam M Nordmann P Mulvey MR Complete nucleotide sequence of a 92-kilobase plasmid harboring the CTX-M-15 extended-spectrum beta-lactamase involved in an outbreak in long-term-care facilities in Toronto, Canada Antimicrob Agents Chemother 2004 48 3758 3764 10.1128/AAC.48.10.3758-3764.2004 15388431 PMC521865

[B49] Lavollay M Mamlouk K Frank T Akpabie A Burghoffer B Redjeb SB Bercion R Gautier V Arlet G Clonal dissemination of a CTX-M-15 β-lactamase-producing Escherichia coli strain in the Paris area, Tunis, and Bangui Antimicrob Agents Chemother 2006 50 2433 2438 10.1128/AAC.00150-06 16801423 PMC1489776

[B50] Cho YJ Moon DC Jin JS Choi CH Lee YC Lee JC Genetic basis of resistance to aminoglycosides in Acinetobacter spp. and spread of armA in Acinetobacter baumannii sequence group 1 in Korean hospitals Diagn Microbiol Infect Dis 2009 64 185 190 10.1016/j.diagmicrobio.2009.02.010 19361944

[B51] Lambert T Gerbaud G Courvalin P Characterization of the chromosomal aac (6′)-Ij gene of Acinetobacter sp. 13 and the aac (6′)-Ih plasmid gene of Acinetobacter baumannii Antimicrob Agents Chemother 1994 38 1883 1889 10.1128/AAC.38.9.1883 7810994 PMC284657

[B52] Shaw K Cramer C Rizzo M Mierzwa R Gewain K Miller G Hare R Isolation, characterization, and DNA sequence analysis of an AAC (6′)-II gene from Pseudomonas aeruginosa Antimicrob Agents Chemother 1989 33 2052 2062 10.1128/AAC.33.12.2052 2515793 PMC172821

[B53] Park CH Robicsek A Jacoby GA Sahm D Hooper DC Prevalence in the United States of aac (6′)-Ib-cr encoding a ciprofloxacin-modifying enzyme Antimicrob Agents Chemother 2006 50 3953 3955 10.1128/AAC.00915-06 16954321 PMC1635235

[B54] Dijkshoorn L Nemec A Seifert H An increasing threat in hospitals: multidrug-resistant Acinetobacter baumannii Nat Rev Microbiol 2007 5 939 951 10.1038/nrmicro1789 18007677

[B55] Perez F Hujer AM Hujer KM Decker BK Rather PN Bonomo RA Global challenge of multidrug-resistant Acinetobacter baumannii Antimicrob Agents Chemother 2007 51 3471 3484 10.1128/AAC.01464-06 17646423 PMC2043292

[B56] Vakulenko SB Donabedian SM Voskresenskiy AM Zervos MJ Lerner SA Chow JW Multiplex PCR for detection of aminoglycoside resistance genes in enterococci Antimicrob Agents Chemother 2003 47 1423 1426 10.1128/AAC.47.4.1423-1426.2003 12654683 PMC152526

[B57] Vanhoof R Godard C Content J Nyssen H Hannecart-Pokorni E Detection by polymerase chain reaction of genes encoding aminoglycoside-modifying enzymes in methicillin-resistant Staphylococcus aureus isolates of epidemic phage types J Med Microbiol 1994 41 282 290 10.1099/00222615-41-4-282 7932622

[B58] Han D Unno T Jang J Lim K Lee S-N Ko G Sadowsky MJ Hur H-G The occurrence of virulence traits among high-level aminoglycosides resistant Enterococcus isolates obtained from feces of humans, animals, and birds in South Korea Int J Food Microbiol 2011 144 387 392 10.1016/j.ijfoodmicro.2010.10.024 21078531

[B59] Montecalvo MA Horowitz H Gedris C Carbonaro C Tenover FC Issah A Cook P Wormser GP Outbreak of vancomycin-, ampicillin-, and aminoglycoside-resistant Enterococcus faecium bacteremia in an adult oncology unit Antimicrob Agents Chemother 1994 38 1363 1367 10.1128/AAC.38.6.1363 8092838 PMC188211

[B60] Leclercq R Enterococci acquire new kinds of resistance Clin Infect Dis 1997 24 S80 S84 10.1093/clinids/24.Supplement_1.S80 8994783

[B61] McKay G Thompson P Wright G Broad spectrum aminoglycoside phosphotransferase type III from Enterococcus: overexpression, purification, and substrate specificity Biochemistry 1994 33 6936 6944 10.1021/bi00188a024 8204627

[B62] Shaw K Rather P Hare R Miller G Molecular genetics of aminoglycoside resistance genes and familial relationships of the aminoglycoside-modifying enzymes Microbiol Rev 1993 57 138 163 8385262 10.1128/mr.57.1.138-163.1993 PMC372903

[B63] Fouhy F Guinane CM Hussey S Wall R Ryan CA Dempsey EM Murphy B Ross RP Fitzgerald GF Stanton C High-throughput sequencing reveals the incomplete, short-term, recovery of the infant gut microbiota following parenteral antibiotic treatment with ampicillin and gentamycin Antimicrob Agents Chemother 2012 56 5811 5820 10.1128/AAC.00789-12 22948872 PMC3486619

[B64] de Vries LE Vallès Y Agersø Y Vaishampayan PA García-Montaner A Kuehl JV Christensen H Barlow M Francino MP The gut as reservoir of antibiotic resistance: microbial diversity of tetracycline resistance in mother and infant PLoS ONE 2011 6 e21644 10.1371/journal.pone.0021644 21738748 PMC3125294

